# Influence of Pomace Matrix and Cyclodextrin Encapsulation on Olive Pomace Polyphenols’ Bioaccessibility and Intestinal Permeability

**DOI:** 10.3390/nu12030669

**Published:** 2020-02-29

**Authors:** Kristina Radić, Bisera Jurišić Dukovski, Dubravka Vitali Čepo

**Affiliations:** Faculty of Pharmacy and Biochemistry, University of Zagreb, Ante Kovačića 1, 10000 Zagreb, Croatia; kradic@pharma.hr (K.R.); bjurisic@pharma.hr (B.J.D.)

**Keywords:** olive pomace extract, cyclodextrin, bioavailability, in vitro gastrointestinal digestion, antioxidant

## Abstract

Olive pomace is a rich source of biologically active compounds, mainly polyphenols. Recently, an efficient and sustainable cyclodextrin (CD)-enhanced extraction was developed. It enabled a relatively simple formulation of high-quality olive pomace extracts (OPEs) that can be used as alternative sources of olive-derived polyphenols in the nutrition and pharma industries. However, biological effects and nutraceutical potential of OPEs are primarily limited by generally low oral bioavailability of major polyphenols (hydroxytyrosol and its derivatives) that can be significantly influenced by OPE matrix and the presence of CDs in formulation. The major goal of this research was to investigate the impact of complex matrix and different types of CDs on gastrointestinal stability and intestinal permeability of major OPE polyphenols, and provide additional data about mechanisms of absorption and antioxidant activity in gut lumen. Obtained results showed high bioaccessibility but relatively low permeability of OPE polyphenols, which was negatively affected by OPE matrix. CDs improved antioxidant efficiency of tested OPEs and tyrosol gastrointestinal stability. Effects of CDs on permeability and the metabolism of particular OPE polyphenols were CD- and polyphenol-specific.

## 1. Introduction

Olive pomace is the residue remaining after olive oil production, consisting mainly of lignin, cellulose, hemicelluloses, residual olive oil, minerals, and phenolic constituents. Polyphenols are meritorious for the negative effects of the olive pomace on both aquatic and terrestrial ecosystems, which is why such waste must be adequately processed before disposal. It has been shown that the polyphenols inhibit plant and bacterial growth, what directly brings to reduction of biodiversity in surface waters and soil [1,2]. On the other hand, olive pomace polyphenols have been in focus of recent research due to possible positive effects on human health, given the wide spectrum of their biological effects [3]. They are mostly phenolic acids, phenolic alcohols, flavonoids, lignans, and secoiridoids. The main compounds related with positive pharmacological effects of the olive pomace are considered to be hydroxytyrosol (HTS) and its derivatives. HTS is a powerful antioxidant with a broad spectrum of biological effects [4] whose potential has been recognized by the European Food Safety Authority (EFSA), which approved a health claim on the effects of olive oil in the year 2011. EFSA confirmed that olive oil polyphenols (standardized to the proportion of HTS and its derivatives) protect LDL particles from oxidative damage [5]. Other health claims concerning effects of olive’s polyphenols are currently being considered, including maintaining the normal concentration of HDL, LDL, triglycerides, and blood glucose, as well as anti-inflammatory, antimicrobic, and photoprotective effects. The reduction of oxidative stress in the intestine is particularly important given that numerous studies have shown that oxidative stress is directly linked to the development of diseases of the GI tract, such as inflammatory bowel disease and tumor colon. The intestine is the site that was shown to be extremely susceptible to oxidative stress. Namely, lipid peroxidation (as a chain reaction that lead to deleterious cell membrane damage) can be initiated by dietary fats.

Considering its phenolic composition, olive pomace is very similar to olive oil and represents an inexpensive and readily available source of HTS derivatives. In the last decade, the potential of the olive pomace as a rich source of biologically active compounds has been recognized in scientific and professional literature, resulting in the development of a large number of effective extraction procedures [6,7]. However, the poor technological properties of raw olive pomace extracts (OPE) limit their wider application as nutraceuticals and food additives [7]. The main prerequisites for obtaining usable OPE are high yield of HTS derivatives, stability, and satisfactory technological properties of dry extracts. In this regard, cyclodextrins (CDs) were successfully applied for achieving higher polyphenol yields and the formulation of stable and organoleptically acceptable olive pomace extracts (OPEs) [7]. Moreover, α-CD, β-CD, and γ-CD are listed on the generally regarded as safe (GRAS) list of the U.S. Food and Drug Administration (FDA) for use as a food additive [8,9,10].

In addition to demonstrating favorable chemical composition and technological properties, the quality of OPE is significantly determined by bioavailability and biological activity of its main active components. Considering OPE’s very similar polyphenolic composition to olive oil, achieving similar pharmacological effects can be expected [11].

However, the biological effects of OPE will be significantly affected by generally reduced bioavailability of olive polyphenols, mostly due to their incomplete intestinal absorption and to rapid biotransformation favoring urinary excretion of olive polyphenols [12]. Some of the major processes that significantly affect their intestinal absorption are matrix depended, and include releasing the active component from the matrix during the digestion process or creating soluble/insoluble complexes with other components during digestion [13,14]. Therefore, data on intestinal absorption of olive polyphenols from oil cannot be extrapolated to significantly different food matrices, such as OPE.

The bioaccessibility of HTS and its derivatives from OPE has rarely been investigated. Recently, Malapert and co-workers [15] investigated the bioaccessibility of HTS from Alperujo in Caco-2 cells, showing generally low availability of HTS, additionally reduced by the Alperujo matrix or concomitant food intake.

Such strong matrix effects on bioaccessibility of biologically active nutraceuticals can be reduced by significant extent by innovative formulation approaches; therefore, the use of pharmaceutical excipients and advanced formulations for oral administration of nutraceuticals has recently been intensified [16].

Among relatively simple but effective approaches is the formulation of nutraceutical-cyclodextrin inclusive complexes. This approach has recently been successfully applied in formulation of OPE-derived extracts and resulted in improved physical-chemical properties of dry extracts, increased shelf life, and higher antioxidant activity in different food- and biological model systems [7,17].

However, the presence of particular CD in OPE can have significant impact on the bioaccessibility and intestinal permeability of OPE polyphenols. CDs are cyclic oligosaccharides used for the improvement of water-solubility and bioavailability of medicinal products. Their key feature is the hydrophobic internal cavity, that provides the ability to form complexes with a variety of guest molecules resulting with the formation of encapsulation complexes [18]. The encapsulation can influence the stability of nutraceutical in gastrointestinal tract by reducing the rate of hydrolysis, oxidation, stearic rearrangement, racemization, enzymatic decomposition, or formation of complexes with other food components which, all together, can have significant impact on nutraceutical bioaccessibility. CDs can also form aggregates with small molecules, influencing their solubility but also their interactions with other molecules in complex matrices [19]. CDs have been successfully applied for enhancement of bioaccessibility of different polyphenol-type nutraceuticals: apigenin [20], resveratrol [21], pomegranate peel polyphenols [22], ellagic acid [23], and ferulic acid [24]. In addition, CDs can influence intestinal permeability of biologically active compounds through their direct impact on biological membranes.

Taking into account various CD effects and the complexity of OPE matrix, it is very hard to predict the exact effect of CDs on polyphenol bioavailability from OPE. Having in mind already proven efficiency of particular CDs on extraction efficiency and functional characteristics of OPE [7,17], the major goal of this research was to investigate the effects of OPE matrix and different types of CDs on gastrointestinal stability and intestinal permeability of OPE-derived hydroxytyrosol, tyrosol, and oleuropein, and provide novel insight into possibilities of CD utilization in formulation of advanced OPE derived nutraceuticals.

## 2. Materials and Methods 

### 2.1. Samples

Olive pomace (OP) was collected from several two-phase mills in Croatia during autumn 2018. OP was kept at −20 °C (Beko CN161220X, Istanbul, Turkey) in polypropylene bags until use. Pre-treatment included drying at 60 °C for 24 h in an incubator (INKO, Zagreb, Croatia), shredding and sieving on Φ 0.8 mm sieve (Prüfsieb DIN 4188, Kassel, Germany), defatting (~ 5 g of the sample was defatting for 2 h with petrol ether) using the Soxhlet apparatus (INKO SK6ESS, Zagreb, Croatia). Polyphenols were extracted from defatted OP without (native sample) or with the addition of the following cyclodextrins: β (bCD), hydroxypropyl β (hpbCD), randomly methylated β (ramCD), and γ (gCD), according to previously optimized procedure [6]. Briefly, OP was mixed with 20% ethanol (20 g/L) and CDs were added in samples as noted above (8 g/L). The extraction was performed on 700 W of microwave power in high performance microwave digestion unit (Milestone 1200 mega, Sorisole, Italy) for 10 min. The extracts were cooled on ice and filtered to remove the crude parts. The listed CDs were chosen according to the number of successful applications of CDs for olive polyphenols encapsulation in literature data. αCD was not included in this study because it was suggested that, regarding its smaller cavity diameter, it would not provide desired complexation of polyphenols. Obtained extracts were dried for 48 h in a lyophilizator (Alpha 1-4 LOC-1, Martin Christ Gefriertrocknungsanlagen GmbH, Osterode am Harz, Germany). In addition, pure main OP polyphenols (hydroxytyrosol (HTS), tyrosol (TS), oleuropein (OLE)) were included in this study in concentration of 40 µg/mL, both as one compound or in a mix. That concentration was chosen to mimic approximately the concentrations in native sample (nat). Stock solutions of pure compounds were prepared in DMSO and diluted with ultrapure water.

### 2.2. Reagents

Petrol ether and dimethyl sulfoxide (DMSO) were purchased from Carlo Erba Reagents while ethanol was from Gram Mol (Zagreb, Croatia). β cyclodextrin (bCD), hydroxypropyl β cyclodextrin (hpbCD), randomly methylated β cyclodextrin (ramCD), and γ cyclodextrin (gCD) were purchased from Wacker–Chemie GmbH (Burghausen, Germany). Methanol (≥ 99.9%) and sodium acetate used for the preparation of chromatographic analysis were from Sigma–Aldrich (St. Louis, MO, USA) while acetonitrile (≥ 99.9%) was from Honeywell (Charlotte, NC, USA), and acetic acid from Kemika (Zagreb, Croatia). Reference standards of phenolic compounds 3-hydroxytyrosol (HTS), tyrosol (TS), and oleuropein (OLE) were of analytical grade (≥ 98%) and purchased from Sigma–Aldrich. 2,2’-azino-bis [3-ethylbenzothiazoline-6-sulphonic acid] (ABTS), potassium persulfate, 6-hydroxy-2,5,7,8-tetramethylchroman-2-carboxylic acid (Trolox), Dulbecco’s Phosphate Buffered Saline (PBS liquid, sterile-filtered, without calcium, without magnesium, suitable for cell culture), and tert-butyl hydroperoxide (tBOOH) were also purchased from Sigma–Aldrich. Bile salts, pancreatin from porcine pancreas (4 × USP), and Dulbecco’s Modified Eagle’s Medium (DMEM with 4500 mg/L glucose, L-glutamine, and sodium bicarbonate, without sodium pyruvate, liquid, sterile-filtered, suitable for cell culture) were purchased from Sigma Aldrich. Rabbit gastric extract (RGE > 25 U/mg) was from Lipolytech (Marseille, France). Heat inactivated fetal bovine serum (FBS), nonessential amino acids (NEAA), penicillin/streptomycin/amphotericin B (A/A), and trypsin were purchased from Capricorn Scientific (Ebsdorfergrund, Germany). 3-[4,5-dimethylthiazole-2-yl]-2,5-diphenyltetrazolium bromide (MTT) was from Panreac AppliChem (Darmstadt, Germany). Ultrapure water (18 MΩ) was obtained from SG Reinstwassersystem Ultra Clear UV Plus coupled with SG Wasservollentsalzer-Patrone SG-2800 (Günzburg, Germany). Acqua pro injectione was obtained from Croatian Institute of Transfusion Medicine. Hank’s balanced salt solution (HBSS) pH 6.0 was prepared by dissolving KCl (0.4 mg/mL), NaHCO_3_ (0.35 mg/mL), NaCl (8.0 mg/mL), D-glucose monohydrate (1.1 mg/mL), KH_2_PO_4_ (0.06 mg/mL), Na_2_HPO_4_×2H_2_O (0.06 mg/mL), CaCl_2_×2H_2_O (0.185 mg/mL), MgCl_2_×6H_2_O (0.1 mg/mL), MgSO_4_×7H_2_O (0.1 mg/mL), and HEPES (7.15 mg/mL) in ultrapure water.

All the solvents needed for chromatographic separation were degassed before analysis with Branson 1210 Ultrasonic Cleaner (Danbury, CT, USA). Acetate buffer was prepared by mixing sodium acetate 0.1 M: acetic acid 0.1 M (2:1 *v*/*v*) and adjusting the pH to 5 with pH meter (702 SM Titrino, Metrohm, Herisau, Switzerland).

### 2.3. Quantification of Phenolic Components by HPLC-FLD and Determination of the Radical Scavenging Capacity by TEAC

The main polyphenols in olive pomace (HTS, TS, and OLE) were identified and quantified by HPLC system (Waters Alliance 2695, Milford, MA, USA) coupled with a 2475 Multi λ detector (FLD) with Xenon lamp, according to slightly modified method of Tsarbopoulos and co-workers [25]. Samples were prepared by filtration through 0.45 µm PES syringe filters (Macherey–Nagel, Düren, Germany). Chromatographic separation was conducted by injecting 20 µL of sample on a reversed phase column (250 × 4.6 mm, 5 µm) (Agilent Zorbax Eclipse Plus C18, Santa Clara, CA, USA). Mobile phases were 0.05 M sodium acetate buffer pH 5 and acetonitrile with the flow rate of 1 mL/min. Elution was conducted over 20 min at 25 °C. Identification was performed with FLD set at the excitation wavelength of 280 nm, and emission wavelength of 316 nm. Polyphenols were identified by comparing the retention times of the eluting peaks with those of the standards. Peaks were quantified by using the Empower2 software (Waters, Milford, MA, USA) and compared to external standard calibration. Standard stock solutions were prepared by dissolving reference compounds in DMSO. Aliquots of these solutions were further diluted with ultrapure water to obtain calibration curve (1–81 mg/L).

Radical scavenging capacity was measured following the procedure for determining Trolox equivalents antioxidant capacity (TEAC) described by Re and co-workers [26]. In brief, ABTS chromophore was generated a day before the experiment through the reaction between ABTS and potassium persulfate. The ABTS˙^+^ was diluted 10× and the initial absorbance was ~ 0.7. Samples were mixed with diluted ABTS˙^+^ and incubated 3 min at 30 °C. Discoloration of the radical was measured at 750 nm using the multimode plate reader (Perkin Elmer Victor X3, Waltham, MA, USA). The percentage of the absorbance decrease was compared to Trolox by using the calibration curve (% ΔA vs. Trolox concentration).

### 2.4. In Vitro Simulated Gastrointestinal Digestion

Bioaccessibility of the OP polyphenols was assessed by in vitro static simulation of gastrointestinal digestion in the upper tract following the standardized protocol described by Brodkorb and co-workers [27]. The procedure was consisted of two sequential incubations; initially in simulated gastric fluid (SGF)/pepsin/gastric lipase to simulated gastric conditions followed by simulated intestinal fluid (SIF)/bile salts/pancreatin to simulate duodenal digestion. Briefly, 500 mg of OPE were dissolved in 2 mL of ultrapure water and mixed with SGF. The samples were incubated at 37 °C for 2 h in a water bath (Büchi B-490, Flawil, Switzerland) with uniform shake at 110 rpm. Then SIF was added and incubated under the same conditions for 2 h. Enzyme solutions were prepared just before use. Samples were put on ice for 10 min and centrifuged (Heraeus Biofuge Stratos, Hanau, Germany) for 20 min at 4 °C and 4100× rpm in order to remove the crude parts of the sample. Supernatants were collected and the enzyme inactivation was done by a heat shock for 5 min at 100 °C in Thermomixer R (Eppendorf, Hamburg, Germany). Then, the samples were cooled in an ice bath for 10 min and centrifuged again under the same conditions. Supernatants, that represent bioaccessible fraction (bf) of the samples, were collected and stored at −80 °C until analyses. Blank sample contained only digestive solutions and was analyzed to discard interferences due to the reagents. The stability of polyphenols during gastrointestinal digestion was monitored by calculating the percentage (%) according to Equation (1).

% _GI_ = (amount in bioaccessible fraction/amount in undigested extract) × 100 (1)

### 2.5. In Vitro Study of Transepithelial Transport

For investigation of transepithelial transport human epithelial colorectal adenocarcinoma cell line (Caco-2) was used. Caco-2 cells (American Type Culture Collection (ATCC)) were cultured in DMEM supplemented with 10% heat-inactivated FBS, 1% NEAA, and 1% A/A. Cell cultures were maintained at 37 °C, in a humidity saturated atmosphere consisted of 5% CO_2_ (Sanyo MCO-20AIC CO_2_ Incubator, Osaka, Japan). Medium was changed every 2 days. Cells were passaged at 80–90% confluence.

The highest non-toxic concentration of bioaccessible OPE fractions and pure polyphenols was determined by MTT assay [28]. 3 × 10^5^ cells/well were seeded in 96-well plates (Thermo Fisher Scientific 130188, Rochester, NY, USA) and grown until reaching confluence (approximately 48 h). The medium was aspirated, and the cells washed with 100 µL PBS/well. Cells were incubated for 2 h with either 100 µL of samples or Hank’s balanced salt solution (HBSS) for positive control. Samples were applied undiluted or diluted (2×, 5×, 10×) with HBSS. Initial concentration of OPE in bioaccessible fractions were 28 mg/mL while HTS, TS, OLE, mix (HTS + TS + OLE) were applied in initial concentration of 40 μg/mL. Samples were then removed, and cells were washed with 100 µL PBS/well. Cell viability was assessed by the addition of 40 µL of MTT 0.5 mg/mL (diluted in PBS) and incubation for 3 h at 37 °C, followed by dissolution of the formazan crystals in 170 µL of DMSO. Absorbance (A) was measured at 490 nm and cell viability was expressed as fold change (FC_v_) relative to the positive control according to Equation (2). Blank absorbance was measured in wells containing MTT without cells.

FC_v_ = (A_sample_ − A_blank_)/(A_control_ − A_blank_) (2)

Transepithelial transport was investigated in 12-well plate with Transwell^®^ permeable supports (Costar 3401, Corning Incorporated, Kennebunk, ME, USA) [29]. The 3 × 10^5^ cells were seeded per well and maintained at 37 °C, in a humidity saturated atmosphere consisted of 5% CO_2_. Cells were grown for 23 days in order to form a differentiated monolayer on filters. The medium was warily aspirated (Gilson Safe Aspiration Station, Middleton, WI, USA) and replaced with fresh one every 2 days. The added volume was 0.5 mL in the apical and 1.5 mL in the basolateral compartment. Monolayer integrity was routinely checked by determining transepithelial electrical resistance (TEER) during the cell growth, before and after the transport experiment. Electrical resistance (ER) was measured with STX2 and EVOM resistance meter (World Precision Instruments Inc, Sarasota, FL, USA). TEER defined as ER per area was calculated according to Equation (3).

TEER = (R − 120 Ω) × 1.12 cm^2^ (3)

where TEER is transepithelial electrical resistance, 120 Ω is resistance of the cell free-well (blank), 1.12 cm^2^ is the filter surface.

On the day of the experiment, the lowest TEER was 228 Ω cm^2^. Day after the experiment, the lowest TEER was 345 Ω cm^2^.

To evaluate transepithelial permeability, medium was aspirated from both apical and basolateral chambers and washed twice with pre-warmed PBS. Then, 0.5 mL of the samples were added to the apical chamber and 1.5 mL of the HBSS to the basolateral chamber of each well. Bioaccessible fractions obtained after the gastrointestinal simulation and pure polyphenols were applied on a cell monolayer in triplicate and incubated at 100 rpm and 37 °C for 2 h in a shaker (Biosan Incubator ES-20/60, Riga, Latvia). The pure analytes (HTS, TS, OLE) were included in this study to examine the potential interference in transepithelial transport among these three polyphenols but also among the other compounds that are present in the OPE. Polyphenols’ content determined by HPLC-FLD and TEAC were determined in apical and basolateral compartment.

After the 2-h incubation of Caco-2 cells with the OPEs and pure polyphenols, transport through the cell monolayer was studied. The content of polyphenols and TEAC were determined in both, apical and basolateral chamber, and expressed as % of the amount applied on cell monolayer according to Equation (4). The difference between the total amounts found in the apical and basolateral chambers at the end of experiment and the initial amount was expressed as metabolized fraction representing the amount that is either metabolized or accumulated inside cells.

% = (amount in apical or basolateral compartment)/initial amount) × 100(4)

### 2.6. Determination of Antioxidative Activity

To assess antioxidant activity of the bioaccessible fractions of OPE and pure polyphenols Caco-2 cell viability was determined. For that purpose, cells were seeded in 96-well microtiter plates at a density of 3 × 10^5^ cells/well in 100 µL of medium and maintained until reaching confluence (approximately 48 h) at 37 °C in a humidity saturated atmosphere consisted of 5% CO_2_. Cells were treated with 20 µL of analyzed OPEs/pure polyphenols for 2 h and then with 20 µL of prooxidant tBOOH for the following 2 h. Final tBOOH concentration was 100 µM. Positive control cells were treated with HBSS only, while negative control cells received HBSS instead of samples and tBOOH. Previously described MTT assay was performed to detect cell viability.

### 2.7. Statistical Analysis

All experiments were run in quadruplicate unless otherwise stated. Data from the bioavailability and antioxidant assays were statistically tested by one-way analysis of variance (ANOVA), followed by Tukey’s multiple comparisons test. Results were expressed as average value and standard deviation. *p* < 0.05 was considered statistically significant unless otherwise noted. GraphPad^®^Prism 6 Software (San Diego, CA, USA) was used for statistical analysis.

## 3. Results and Discussion

### 3.1. Composition of Olive Pomace Extracts

**Figure 1:**
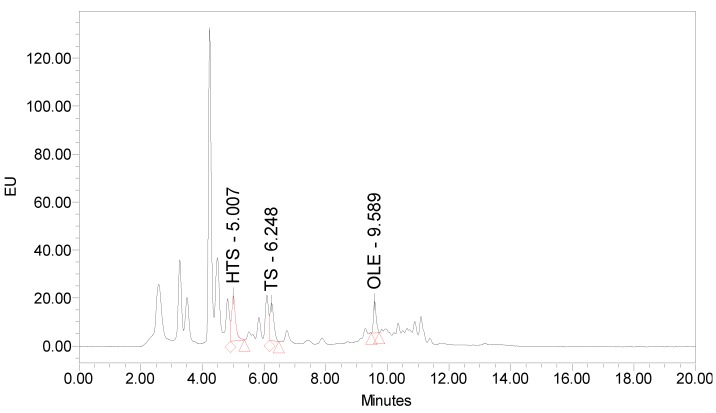
Chromatogram of the native olive pomace extract.

The amount of main polyphenols in olive pomace (HTS, TS, and OLE) and TEAC are noted in Table 1. Polyphenols were identified by comparing the retention times of the eluting peaks (Figure 1) with those of the standards. Peaks were quantified by comparison to external standard calibration curve. TEAC was calculated as the percentage of the absorbance decrease compared to Trolox by using the calibration curve (% ΔA vs. Trolox concentration).

### 3.2. Bioaccessibility of Olive Pomace Polyphenols

Bioaccessibility is defined as the quantity of a compound that is released from food matrix in gastrointestinal tract, becoming available for absorption [30]. Since it is known that it can be greatly dependent on the type of food matrix [31], we took into account that the presence of different cyclodextrins in analyzed OPE formulations might influence the bioaccessibility of target compounds.

**Figure 2:**
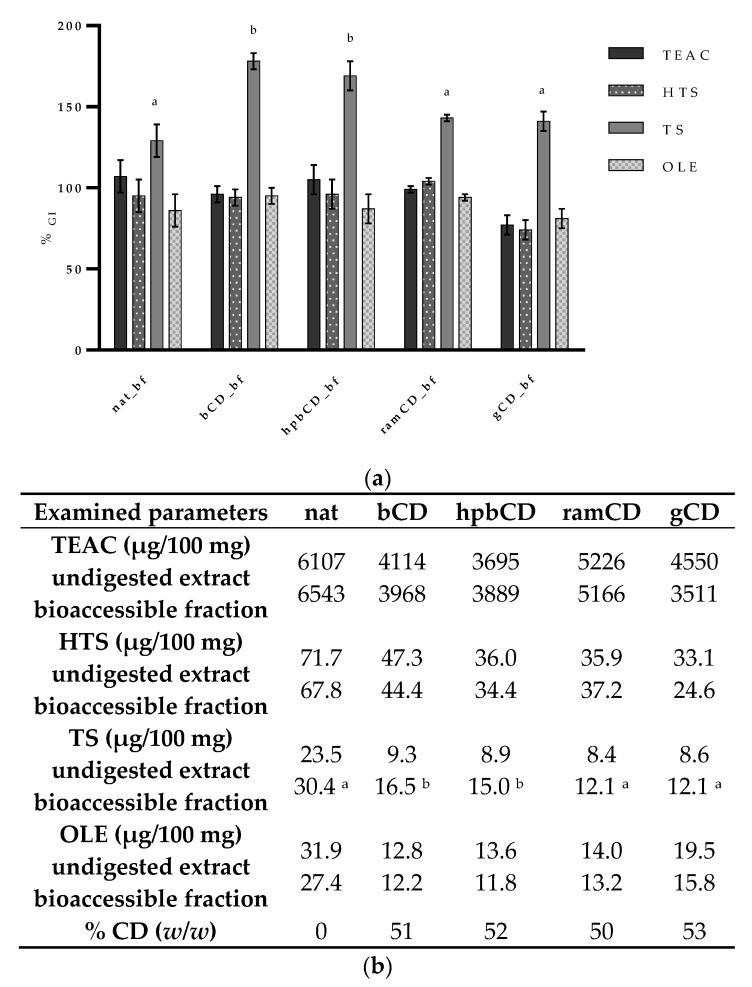
Bioaccessibility of total antioxidants and main polyphenols present in OPEs. (**a**) Relative change expressed as percentage of each component remained after the in vitro simulated GI digestion. Data are presented as mean ± standard deviation (*n* = 3). (**b**) Absolute values of concentrations (*w*/*w*) in undigested samples and bioaccessible fractions; ^a^ indicate the significant difference of percentage among all samples in each group (*p* < 0.004); ^b^ indicates the significant difference of percentage among all samples in each group (*p* < 0.0001); TEAC (Trolox equivalent antioxidant capacity), HTS (hydroxytyrosol), TS (tyrosol), OLE (oleuropein), OP (olive pomace), bf (bioaccessible fraction), CD (cyclodextrin), nat (native OP), bCD (OP + β CD), hpbCD (OP + hydroxypropyl β CD), ramCD (OP + randomly methylated β CD), gCD (OP + γ CD).

The impact of gastrointestinal conditions on the bioaccessibility of total antioxidants and particular phenolic compounds is presented in Figure 2. Results showed that both, antioxidative capacity and the amounts of HTS, TS, and OLE remain constant during the gastrointestinal digestion. The only change that was observed was an increase of TS amount in all samples’ bioaccessible fractions. Considerable increase of TS amount in bioaccessible fraction was also noted by Corona and co-workers [13] after 2-h incubation of olive oil in HCl. Interestingly, in the same work, that phenomenon was observed also for HTS unlike in our work indicating specific matrix effect, which is in agreement with available literature data [32]. Good GI stability of HTS, shown in our work, has already been reported by several authors [15,33,34].

It is important to emphasize that both, HTS and TS can be products of disintegration of more complex polyphenols present in olive pomace in acidic conditions, i.e., secoiridoids or verbascosides [35]. The increase of the amount of TS was probably due to the liberation from verbascosides [36] and it was additionally modified by the presence of particular CDs in the formulation. Our results showed that ramCD and gCD did not influence the release of TS in OPE while the presence of bCD and hpbCD caused significant increase of TS release rate during the GI digestion. More precisely, after digestion of nat, ramCD, and gCD, relative change of TS was 129–143%, while TS amount in bCD_bf and hpbCD_bf increased to 169–178% comparing to the undigested sample. Observed changes might be explained by the strong binding capacity of bile salts that can interact with phenolic hydroxyl groups to form hydrogen/ionic bonds [37]. Formation of inclusion complexes or aggregates of TS with bCD and hpbCD [38] might prevent adsorption resulting in increased bioavailability. Despite the significant increase of TS amount in all samples, their antioxidative potential did not change significantly which could be a confirmation of its weak antioxidant potential. However, TS was shown to be an effective cellular antioxidant, probably due to its intracellular accumulation [39].

The amount of the main olive secoiridoid (OLE) after gastrointestinal digestion was the same as in undigested extracts. This is in accordance to available literature data [40] and the fact that glycosylated secoiridoids are not to be subjected to gastric hydrolysis [34].

Our results clearly show that the main polyphenols and antioxidants from OPE do not degrade under conditions of gastrointestinal digestion and that TS bioaccessibility varies depending on the type of CD used for encapsulation. In comparison to data obtained for olive oil [11,41], gastrointestinal availability of HTS, TS, and OLE from OPE is comparable or higher, indicating that olive pomace matrix doesn’t impair bioaccessibility of target phenolic compounds, confirming the applicability of OPE as potential alternative food source of olive polyphenols.

### 3.3. Permeability of OPE Polyphenols on Caco-2 Cell Monolayer

**Figure 3:**
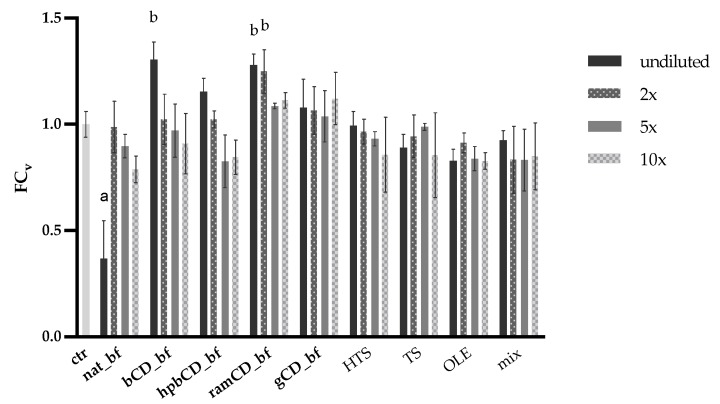
Effect of a 2-h exposure of olive pomace extracts (OPEs’) bioaccessible fractions and pure polyphenols on Caco-2 cell viability. Control cells (ctr) were treated with Hank’s balanced salt solution (HBSS). Data are presented as mean ± standard deviation of the fold change calculated according to Equation (2); ^a^ significant decrease of cell viability compared to ctr (*p* < 0.001); ^b^ significant increase of cell viability compared to ctr (*p* < 0.001); HTS (hydroxytyrosol), TS (tyrosol), OLE (oleuropein), mix (HTS + TS + OLE) in initial concentration of 40 μg/mL; bf (bioaccessible fraction), CD (cyclodextrin); nat (native OP), bCD (OP + β CD), hpbCD (OP + hydroxypropyl β CD), ramCD (OP + randomly methylated β CD), gCD (OP + γ CD) in initial concentration of 28 mg/mL. 2×, 5×, and 10× are dilutions of the samples.

The potential cytotoxic effect of OPEs’ bioaccessible fractions and pure polyphenols (HTS, TS, OLE, and their mix) was investigated to ensure the integrity of the cell monolayer. Their influence on the viability of human epithelial colorectal adenocarcinoma cells Caco-2 was determined by using the MTT assay. Cells were treated with either undiluted samples (bioaccessible fraction of OPEs in initial concentration of 28 mg/mL or HTS, TS and OLE in initial concentration of 40 µg/mL) or the same samples diluted 2×, 5× or 10× with HBSS. The results presented in Figure 3 showed that cell viability was significantly decreased when cells were treated with undiluted nat_bf. The possible explanation for this toxic effect is the fact that polyphenols may exert a pro-oxidant effect at high doses [42,43]. Cell viability was increased after the treatment with undiluted bCD_bf and ramCD_bf, and 2× diluted ramCD_bf. Treatments with all the other samples did not affect the Caco-2 viability. Based on these findings, nat_bf was 2× diluted with HBSS for further investigations, so its initial concentration was 14 mg/mL. Accordingly, mix was diluted 2× for comparison with nat and the initial concentration of each polyphenol was 20 µg/mL.

The first goal of permeability research was to evaluate the potential interference between HTS, TS, and OLE transport through Caco-2 monolayer. For that purpose, tested compounds were applied to Caco-2 monolayer as pure compounds and as a mixture (mix). Both, apical to basolateral (a–b), and basolateral to apical (b–a) transport was investigated in order to elucidate the transport mechanisms of each tested compound. To determine the amount of the compounds transported per time, the apparent permeability coefficient (P_app_) was calculated according to the following general equation that does not require sink conditions:

(5) $$C_{R}\left(t\right)=\left(\frac{M}{V_{A}+V_{B}}\right)+\left(C_{R,0}-\frac{M}{V_{A}+V_{B}}\right)e^{-P_{\mathrm{app}}A\left(\frac{1}{V_{A}}+\frac{1}{V_{B}}\right)t}$$

where V_A_ is the volume in the apical compartment, V_B_ is the volume of the basolateral compartment, A is the area of the filter (1.12 cm^2^), M is the total amount of substance in the system (mol), C_R,0_ is the concentration of the substance in the basolateral compartment at the start of the time interval and C_R(t)_ is the concentration of the substance at time t measured from the start of the time interval (s).

P_app_ of hydroxytyrosol was approximately 1 × 10^−5^ cm s^−1^ while P_app_ of tyrosol was three times higher (approximately 3 × 10^−5^ cm s^−1^). There was no difference in P_app_ when TS was added to the apical or basolateral chamber indicating a passive transport through the monolayer. The efflux ratio (defined as the quotient of the secretory permeability and the absorptive permeability (P_app_b__–a_/P_app_a__–b_)), and the uptake ratio (the inverse of the efflux ratio, P_app_a__–b_/P_app_b__–a_) results are available in Table S1. There are two studies in which P_app_ of TS was determined [13,44]. Interestingly, these two studies obtained significantly different results. Our was in accordance to work from Corona and co-workers [13] and the P_app_ value was approximately 20 times higher than the one from D’Antuono and co-workers [44]. The possible reason for that significant difference is that in the mentioned study, Caco-2/TC7 cells were used for permeability examination. Caco/TC7 are characterized with more developed intercellular junctions. Those junctions act as diffusion barriers within the lipid bilayer of the plasma membrane which can decrease the rate of passive transport. HTS permeability, on the other hand, was studied by several authors [13,45,46]. P_app_ of approximately 1 × 10^−5^ cm s^−1^ we obtained for HTS was in accordance with all these studies. However, we also observed that HTS is effluxed back to the apical chamber with the ratio of 1.47 ± 0.07 (data available in supplementary material). P_app_ of OLE was determined only when present along with HTS and TS (mix). However, the value was 1 × 10^−7^ cm s^−1^ indicating very low permeability of OLE what indicates its intensive metabolism in the Caco-2 cells.

**Figure 4:**
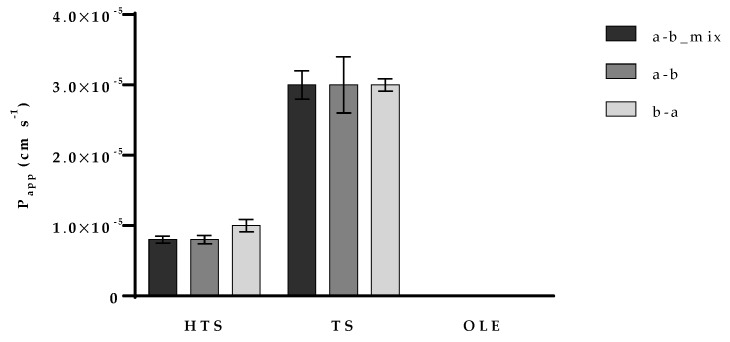
Determination of the hydroxytyrosol (HTS) olive pomace polyphenols’ transport mechanism. P_app_ (apparent permeability coefficient) was determined for HTS (hydroxytyrosol), TS (tyrosol), and OLE (oleuropein) transport from apical (a) to basolateral (b) chamber either in their mix (a–b_mix) or as one-compound (a–b), and when transported from basolateral to apical chamber (b–a). Data are presented as mean ± standard deviation. All the experiments were done in triplicates.

Obtained results also showed that there was no difference between P_app_ of HTS and TS obtained by testing one-compound solutions or the mix (Figure 4) indicating no interference in their absorption. OLE, on the other hand, showed slightly lower metabolism rate when present along with HTS and TS. As far as we know, there are no published studies of the potential permeability interference of olive polyphenols and therefore we find this result an important prerequisite for the development of olive-derived nutraceuticals.

**Figure 5:**
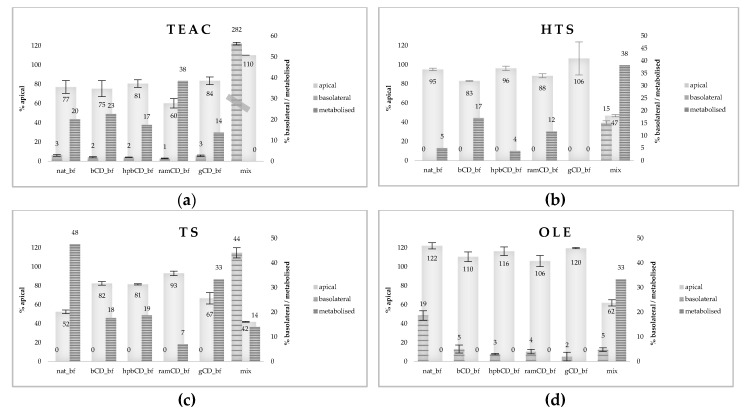
Permeability of total antioxidants (**a**) and specific polyphenols (HTS (**b**), TS (**c**), and OLE (**d**)) from OPEs bioaccessible fractions and mix. Polyphenols’ amount and TEAC (Trolox equivalent antioxidant capacity) were determined in both, apical and basolateral chamber and expressed as percentage of the amount applied on cell monolayer. The difference between the amount found in the apical and basolateral chamber and the initial amount was expressed as metabolized fraction representing the amount that is either metabolized or accumulated inside cells. Initial concentrations were: 14 mg/mL for nat (native OP); 28 mg/mL for bCD (OP + β CD), hpbCD (OP + hydroxypropyl β CD), ramCD (OP + randomly methylated β CD), gCD (OP + γ CD); 20 μg/mL for mix (hydroxytyrosol, tyrosol, oleuropein); data are presented as mean ± standard deviation. All the experiments were done in triplicate; OPE (olive pomace extract), bf (bioaccessible fraction), CD (cyclodextrin).

In the second phase of investigation we determined the permeability of total antioxidants, HTS, TS, OLE, and from OPEs bioaccessible fractions (Figure 5). Permeability of total antioxidants was assessed based on investigation of TEAC and it revealed a significant decrease of the antioxidative potential in the apical chamber of all investigated OPEs while TEAC in the basolateral chambers was rather low, from 1% to 3% of the value applied to cell monolayer (Figure 5a). Obtained results indicate high permeability, but also extensive metabolism of OPE-derived antioxidants.

However, results were significantly different for mix (pure HTS+TS+OLE) where we noticed the slight increase in TEAC in the apical but also 2.82 times increase in TEAC in the basolateral compartment, indicating high bioavailability. Obtained results indicate that OPE matrix decreases bioavailability of antioxidants and that bioavailability of HTS, TS, and OLE is increased when they are applied as the mixture of pure compounds. Additionally, results indicate “formation” of antioxidants during transport through Caco-2 monolayer, when they are applied as mixture of pure compounds. The explanation of this phenomena could be the fact that glucoside residue of OLE in mix is removed by the small intestine brush border or cytosolic enzymes resulting with increased antioxidant activity and increased permeability of the aglycone compound. The deglycosylation of dietary flavonoid glycosides was studied in detail by Németh and co-workers [30], where two β-glucosidases were isolated from human small intestine mucosa: lactase-phlorizin hydrolase (localized to the apical membrane of small intestinal epithelial cells) and cytosolic β-glucosidase; they significantly affected flavonoids’ absorption and metabolism. Permeability of OLE from mix is low, and metabolism extensive (as shown in Figure 4). Therefore, we can assume that in Caco-2 cells OLE and OLE aglycone were metabolized into potent antioxidants, such as HTS, resulting in significant increase in TEAC in basolateral chamber. In OPE samples the effect is absent probably due to saturation of brush border and cytosolic enzymes by other substrates present in complex matrix.

The explanation of TEAC results is confirmed by data obtained for OLE permeability (Figure 5d). OLE content in apical chambers increased during 2 h of incubation, particularly in nat_bf. Since it was shown that lactase-phlorizin hydrolase has low but significant ability to hydrolyze several natural β-glycosides (also cellulose), it could be that the part at the OLE entrapped in the OP matrix was liberated during incubation and that this reaction was inhibited by the presence of CDs in reaction mixture. In contrast, in mix (pure HTS+TS+OLE), OLE apical content was decreased to 62% of initial concentration indicating formation of OLE aglycone, as previously explained. However, extensive metabolism of mix resulted in low basolateral OLE, which was comparable to that of CD-encapsulated OPEs. The highest bioavailability of OLE was from native sample, indicating negative effect of CDs on OLE permeability.

HTS and TS were not found in basolateral chambers, except when present as a mix of pure analytes (15% and 44% of the initial amount respectively) indicating strong negative matrix effect on their in vitro bioavailability. On the other hand, significant decrease of their amount in apical chambers indicates extensive metabolism. Figure 5b shows that amount of metabolized/accumulated HTS fraction was increased in bCD_bf and ramCD_bf (17%, 12%, respectively) signaling, enhancing effect of bCD and ramCD on HTS metabolism rate and/or permeation in the cell.

According to available literature data, HTS is transported by passive diffusion, and the transport is bi-directional [46]. Negative effect of OPE matrix on HTS permeability might be explained by non-covalent polyphenol/macromolecule interactions that are largely due to weak associations (combination of hydrogen bonds and hydrophobic interactions) and can impair the possibility of passive diffusion through intestinal epithelium [47]. On the other hand, presence of bCD and ramCD in reaction mixture increased the permeability of HTS, probably due to their previously reported ability to increase the permeability of Caco-2 monolayer by removing cholesterol from the membranes changing their structure and resulting in higher permeability [48]. Unchanged amount of HTS in apical compartments of analyzed OPEs can be explained by the fact that most polyphenols found in natural matrices exist as esters, glycosides or polymers that can be hydrolyzed and release aglycons at the brush border of small intestine epithelial cells due to the activity of brush-border hydrolases [49].

Permeation of TS inside the cell was significantly higher in comparison to HTS, resulting in decreased apical fraction in all samples except ramCD_bf (Figure 5c). Obtained results are consistent with available literature data indicating higher relative transport rate (apical to basolateral) of TS in comparison to HTS [11]. Generally, permeation of TS was negatively influenced by the presence of all investigated CDs, resulting in lower metabolized fraction in comparison to nat_bf. ramCD_bf showed particularly strong effect and completely blocked the permeation of TS through apical membrane. TS was not detected in basolateral departments of analyzed OPEs probably due to intensive metabolism in Caco-2 cells, which is consistent with available literature data [50]. The amount of TS that might have remained inside the Caco-2 cells was the highest in nat_bf and gCD_bf (48% and 33%, respectively). Since TS is known as an effective cellular antioxidant [39], nat_bf and gCD_bf could be proposed as potent sources of TS which exert its effect after the accumulation in the cells. Data obtained for mix show much higher permeability of TS in comparison to OPE indicating negative effect of matrix on TS bioavailability. Observation is consistent to available literature data and can be explained by the same mechanisms as in the case of HTS [47].

### 3.4. Antioxidative Effect of OPE on Caco-2 Cells

**Figure 6:**
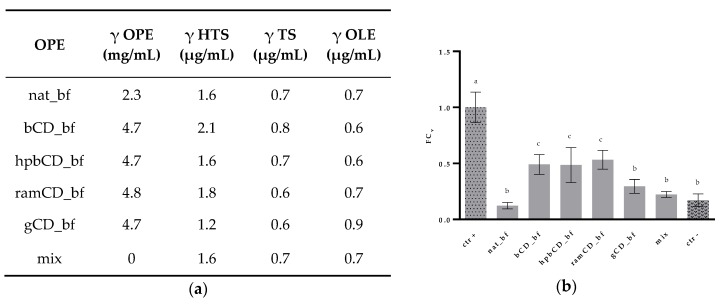
Determination of antioxidative effect of OPEs bioaccessible fractions and mix of polyphenols. (**a**) mass concentration and polyphenol composition of each OPE administered in each well with confluent Caco-2 monolayer. (**b**) Caco-2 cell viability determined by the MTT assay when treated with HBSS (ctr+); OPE and tBOOH (nat_bf, bCD_bf, hpbCD_bf, ramCD_bf, gCD_bf); HTS, TS, and OLE, and tBOOH (mix); and tBOOH (ctr-). Cell viability was expressed as fold change calculated by Equation (2). Data was expressed as mean ± standard deviation. All the experiments were done in 3 replicates; HTS (hydroxytyrosol), TS (tyrosol), OLE (oleuropein), mix (HTS + TS + OLE), bf (bioaccessible fraction), CD (cyclodextrin), nat (native OP), bCD (OP + β CD), hpbCD (OP + hydroxypropyl β CD), ramCD (OP + randomly methylated β CD), gCD (OP + γ CD), OPE (olive pomace extract), tBOOH (tert-butyl hydroperoxide).

To assess the antioxidative effect of OPEs we determined their effect in decreasing oxidative stress in small intestine epithelial cells. Cells were treated with OPE in concentrations noted in Figure 6a. Mix was prepared with HTS, TS, and OPE in the same amounts as in nat_bf. All of the samples contained similar amounts of these polyphenols, i.e., HTS 1.2–2.1, TS 0.6–0.8, and OLE 0.6–0.9 μg/mL. The treatment of Caco-2 cells with 100 µM tBOOH significantly reduced cell viability clearly showing the toxic effect of peroxide. Pre-incubation with mix did not show protective effect against prooxidant in this high concentration as well as nat_bf and gCD_bf. However, we observed a significant increase in viability at cells treated with bCD_bf, hpbCD_bf, and ramCD_bf (Figure 6b). The capability of CDs to improve the biological, chemical and physical properties of plant bioactive molecules was observed by many authors [51]. Particularly their ability to improve the solubility of the bioactive compounds is important since it is directly connected to the exertion of biological activities. It could be that other compounds in OPE that possess antioxidative activity can exert their potential when encapsulated by bCD, hpbCD, and ramCD.

## 4. Conclusions

In this work we evaluated the influence of OPE matrix and CDs on bioaccessibility and intestinal permeability of the main olive pomace polyphenols. Obtained results indicate that major olive pomace polyphenols are stable during gastrointestinal digestion. Presence of CDs in formulation significantly increases bioaccessibility of TS, probably by formation of inclusion complexes and prevention of TS adhesion to bile salts or other macromolecules present in reaction mixture during simulation of OPE digestion. Investigation of permeability of pure polyphenols (HTS, TS, and OLE) showed that HTS and TS are primarily absorbed by passive diffusion and confirmed extensive intracellular metabolism of OLE in Caco-2 cells. P_app_ was unaffected when HTS and TS were applied as a mix, indicating the absence of interactions at the absorption level. OPE matrix negatively influenced the permeability of OPE total antioxidants and investigated polyphenols. However, observed effects were comparable to those of olive oil reported by other authors. Presence of CDs in tested formulations significantly affected permeability of particular polyphenols but results were CD- and polyphenol specific. bCD and ramCD increased permeability of HTS, probably through direct effects on Caco-2 cell monolayer. gCD significantly increased intracellular TS concentrations. Impact of CDs on OLE permeability was generally negative, but to a much lesser extent in comparison to matrix effect. Investigation of antioxidative activity showed that OPE can be considered a rich source of highly permeable antioxidants other than HTS, TS and OLE. OPE also provided efficient protection of Caco-2 cells against oxidative stress, and their antioxidative activity was positively affected particularly by bCD, hpbCD and ramCD.

## Figures and Tables

**Table 1:** Olive pomace extracts’ composition.

OPE	HTS (µg/100 mg)	TS (µg/100 mg)	OLE (µg/100 mg)	TEAC (µg/100 mg)	% CD (*w*/*w*)
nat	71.7	23.5	31.9	6107	0
bCD	47.3	9.3	12.8	4114	51
hpbCD	36.0	8.9	13.6	3695	52
ramCD	35.9	8.4	14.0	5226	50
gCD	33.1	8.6	19.5	4550	53

## Supplementary Materials

The following are available online at https://www.mdpi.com/2072-6643/12/3/669/s1, Table S1: Determination of the HTS olive pomace polyphenols’ transport mechanism.
