# Supplementary material for: Influence of Pomace Matrix and Cyclodextrin Encapsulation on Olive Pomace Polyphenols’ Bioaccessibility and Intestinal Permeability
Source: Nutrients. 2020 Feb 29;12(3):669. doi: 10.3390/nu12030669 (PMC7146296; doi:10.3390/nu12030669)
Supplement: Supplementary file 1 [file nutrients-12-00669-s001.pdf]

Table S1. Determination of the HTS olive pomace polyphenols' transport mechanism.

Transport	P_{app} (cm s^{-1})		
	**HTS**	**TS**	**OLE**
a–b_mix	8.1E-06 ± 4.8E-07	3.2E-06 ± 2.4E-06	1.1-07 ± 1.1-08
a–b	7.6E-06 ± 6.3E-07	3.2E-06 ± 3.5E-06	0
b–a	1.2E-05 ± 8.6E-07	3.0E-06 ± 8.9E-07	0
Ratio			
Efflux	1.47 ± 0.07	0.93 ± 0.00	N/A
Uptake	0.67 ± 0.03	1.08 ± 0.00	N/A

Papp (apparent permeability coefficient) was determined for HTS (hydroxytyrosol), TS (tyrosol), and OLE (oleuropein) transport from apical (a) to basolateral (b) chamber either in their mix (a–b_mix) or as one-compound (a–b), and when transported from basolateral to apical chamber (b–a). The efflux ratio is defined as the quotient of the secretory permeability and the absorptive permeability (Papp_b–a/Papp_a–b). Uptake ratio is defined as the inverse of the efflux ratio (Papp_a–b/Papp_b–a). Data are presented as mean ± standard deviation. All the experiments were done in triplicates.
